# Influence of the modifiers in polyol method on magnetically induced hyperthermia and biocompatibility of ultrafine magnetite nanoparticles

**DOI:** 10.1038/s41598-023-34738-z

**Published:** 2023-05-15

**Authors:** Adrian Radoń, Agnieszka Włodarczyk, Łukasz Sieroń, Magdalena Rost-Roszkowska, Łukasz Chajec, Dariusz Łukowiec, Agnieszka Ciuraszkiewicz, Piotr Gębara, Stanisław Wacławek, Aleksandra Kolano-Burian

**Affiliations:** 1 Faculty of Mechanical Engineering, Silesian University of Technology, Konarskiego 18 a St., 44-100 Gliwice, Poland; 2 Łukasiewicz Research Network - Institute of Non-Ferrous Metals, Sowinskiego 5 St, 44-100 Gliwice, Poland; 3 Department of Medical Genetics, Faculty of Medical Sciences in Katowice, Medical University of Silesia, Medyków 18, 40-752 Katowice, Poland; 4 Institute of Biology, Biotechnology and Environmental Protection, University of Silesia in Katowice, Bankowa 9, 40-007 Katowice, Poland; 5 Department of Physics, Częstochowa University of Technology, Armii Krajowej 19, 42-200 Czestochowa, Poland; 6 Institute for Nanomaterials, Advanced Technologies and Innovation, Technical University of Liberec, Studentská 1402/2, 461 17 Liberec 1, Czech Republic

**Keywords:** Cell biology, Cell death, Cancer, Magnetic properties and materials, Nanoparticles, Synthesis and processing, Biomedical engineering

## Abstract

Magnetite nanoparticles (Fe_3_O_4_ NPs) are widely tested in various biomedical applications, including magnetically induced hyperthermia. In this study, the influence of the modifiers, i.e., urotropine, polyethylene glycol, and NH_4_HCO_3,_ on the size, morphology, magnetically induced hyperthermia effect, and biocompatibility were tested for Fe_3_O_4_ NPs synthesized by polyol method. The nanoparticles were characterized by a spherical shape and similar size of around 10 nm. At the same time, their surface is functionalized by triethylene glycol or polyethylene glycol, depending on the modifiers. The Fe_3_O_4_ NPs synthesized in the presence of urotropine had the highest colloidal stability related to the high positive value of zeta potential (26.03 ± 0.55 mV) but were characterized by the lowest specific absorption rate (SAR) and intrinsic loss power (ILP). The highest potential in the hyperthermia applications have NPs synthesized using NH_4_HCO_3_, for which SAR and ILP were equal to 69.6 ± 5.2 W/g and 0.613 ± 0.051 nHm^2^/kg, respectively. Their application possibility was confirmed for a wide range of magnetic fields and by cytotoxicity tests. The absence of differences in toxicity to dermal fibroblasts between all studied NPs was confirmed. Additionally, no significant changes in the ultrastructure of fibroblast cells were observed apart from the gradual increase in the number of autophagous structures.

## Introduction

Magnetite nanoparticles are one of the most promising nanomaterials in medical applications according to their unique physicochemical properties and biocompatibility^1,2^. Additionally, Fe_3_O_4_ NPs can be synthesized in various sizes, shapes and in the form of core–shell structures, in which shells can be inorganic or polymer-based^3–5^. The many synthesis methods and modification protocols were proposed in the literature to synthesize even multifunctional platforms dedicated to nanomedicine. Furthermore, various factors, not only morphological ones, such as doping and surface functionalization, change the properties and modify the range of applicability of magnetite nanoparticles. Despite their possible use in medicine as MRI contrast, drug delivery systems, anticancer agents, and hyperthermia^6,7^, their application range is much broader and includes catalysis^8^, heavy metals adsorption^9^, microwave absorption^10^, and supercapacitors^11^.

Modifying magnetite nanoparticles' morphology and surface chemical composition can be performed during the synthesis stage and afterward. Roca et al.^3^ have shown that the shape of the nanoparticles can be controlled in a few ways, including modifying the iron source precursors and using selected organic modifiers. Moreover, the size and spontaneous functionalization of the magnetite nanoparticle surface can be controlled in the co-precipitation method by using various organic modifiers such as dextrin and organic acids (tartaric and citric ones)^12^. While the shape and size of Fe_3_O_4_ NPs influence both biocompatibility and magnetically induced hyperthermia effect, the functionalization of their surface allows the synthesis of hydrophobic or hydrophilic nanoparticles^13–16^. Generally, magnetite nanoparticles should be hydrophilic in biomedical applications to form a stable water-based dispersion. To achieve that, the magnetite surface can be refunctionalized, or co-precipitation and polyol methods should be chosen to synthesize nanoparticles with high colloidal stability^17–19^. While the co-precipitation method is one of the most studied ones with high synthesis yield, the prepared nanoparticles are agglomerated, and their size distribution is wide. Accordingly, the polyol method is most promising in biomedical applications. In this case surface of nanoparticles can be functionalized by the reducing solvent^19^ or by introducing into the reaction solution organic modifiers such as ethylenediamine, (3-aminopropyl) triethoxysilane, and citric acid^20–22^. Functionalized magnetite nanoparticles can be used then as MRI contrast or as the agent in magnetically induced hyperthermia.

Magnetic NPs show great potential for biomedical applications^23^. One of them is the induction of local hyperthermia to destroy a particular type of cells and tissues. In this case, magnetite nanoparticles can be used as an agent for targeted therapy, in which NPs are introduced into the tumor and hyperthermia is induced to destroy these tumor cells while other organs and tissues remain intact^24^. Compared to broadly used chemotherapy with many severe side effects, this is a significant advantage. However, for them to be efficiently used in anticancer therapy, NPs should possess two main properties. One is the ability to induce hyperthermia strong enough to kill tumor cells in response to magnetic stimuli, and second, their use should be safe and cause no other side effects.

Unfortunately, due to their small size, NPs can potentially appear toxic to healthy cells and tissues where hyperthermia is not induced^25^. Moreover, in the presence of enzymes in biological structures, NPs can undergo chemical and structural changes that alter their biological properties^26^. Thus their toxic effect can appear later and affect tissues sometimes after treatment. Therefore, it is essential to synthesize NPs as less toxic as possible. Many factors influence NPs toxicity, such as composition, size, aggregate tendency, and surface modification^25,26^. Coating with organic and nonorganic compounds such as polyethylene glycol and polyvinyl alcohol is the most common modification of NPs that decreases their toxicity^27^. This modification prevents the aggregation of NPs and protects them from interaction with proteins, enzymes, and other cell compounds^28^. Another type of modification is functionalization to give them appropriate chemical and physical properties, e.g., electric charge^27^. Also, combination with organic compounds or proteins affects NPs internalization and toxicity^29^. Moreover, coupling with proteins can give them affinity to particular types of cells (desirable for NP-mediated targeted drug delivery) or increase their biocompatibility^30^.

The literature shows that magnetite nanoparticles show low cytotoxicity on human fibroblasts^31^; however, treatment of patients with magnetite nanoparticles may undoubtedly have a negative impact on some cells and lead to the development of diseases such as Parkinson's disease, Alzheimer's disease^32,33^ or cardiovascular diseases^34^. It is known that the cytotoxicity of nanoparticles depends on aggregation rate, environmental conditions, size of nanoparticles, shape, concentration, proportion, and type of polymers used to form the shell^33^. Therefore, researches on nanoparticles are crucial to improve their performance without losing their biocompatibility, thus minimizing the possibility of developing diseases related to their use during treatment. Accordingly, in this study, ultrafine magnetite nanoparticles with a size of about 10 nm and modified surface were synthesized using the polyol method. Organic (polyethylene glycol, hexamethylenetetramine) and inorganic (ammonium bicarbonate) modifiers were added to determine the role of the synthesis method and modification on the surface chemistry and magnetically induced hyperthermia effect. While polyethylene glycols with various molecular weights are widely tested in the synthesis of magnetite nanoparticles dedicated to biomedical applications, ammonium bicarbonate and hexamethylenetetramine were not yet tested in this field; however, some of the works confirm their role in the synthesis of magnetite nanoparticles (especially using hydrothermal route) with unique morphology and properties^35–38^. Accordingly, the role of these modifiers on the size and colloidal stability was examined, together with the possibility of applying magnetite nanoparticles in cyclic hyperthermia treatment, which could be an interesting approach in the controlled drug delivery process in anticancer treatment. Furthermore, their toxicity to human fibroblast cells in vitro was tested and discussed in the context of the applied synthesis modifications.

## Materials and methods

### Magnetite nanoparticles synthesis

Magnetite nanoparticles were synthesized using the polyol method and polyethylene glycol 600 (PEG), urotropine (hexamethylenetetramine), and NH_4_HCO_3_ as modifiers. The synthesized samples were marked as Fe_3_O_4_—PEG, Fe_3_O_4_—URO, and Fe_3_O_4_—NH_4_HCO_3_, respectively. To synthesize Fe_3_O_4_—URO and Fe_3_O_4_—NH_4_HCO_3_ nanoparticles, five mmol of Fe(acac)_3_ was dissolved in 100 ml of triethylene glycol (TREG). Afterward, two mmol of hexamethylenetetramine or NH_4_HCO_3_ was added to the solution and heated to 271 °C. The synthesis was carried out in 30 min. Next, the product was cooled to room temperature, and 100 ml of ethyl acetate was added to precipitate ultrafine nanoparticles. Finally, the black product was removed from the post-reaction solution using a magnetic field and washed thrice with ethyl acetate. A similar procedure was applied to obtain Fe_3_O_4_—PEG nanoparticles. In this case, synthesis was performed using PEG and TREG (25:75 ml, respectively) without adding other modifiers. The synthesized nanoparticles, for further characterization, were stored in ethyl acetate. Before all tests, nanoparticles were removed from the ethyl acetate using a magnetic field. Afterwards, samples were washed with the DI water three times and dried at 60 °C to ensure that ethyl acetate was not presented in dispersions dedicated to the magnetic hyperthermia and cytotoxicity measurements.

### Structure characterization

The structure and phase purity of synthesized samples were analyzed using the X-ray diffraction method. The investigation was carried out on a Rigaku MiniFlex 600 (Rigaku Corporation, Tokyo, Japan) diffractometer equipped with copper tube Cu Kα(λ = 0.15406 nm) as a radiation source (tube voltage 40 kV, current 15 mA). The scan step width was 0.02° in the scan range from 20° to 90° of the 2θ scale. The morphology and structure of synthesized samples were determined using a transmission electron microscope (TEM) S/TEM TITAN 80–300 (FEI Company, Eindhoven, The Netherlands). For this purpose, a few milligrams of the synthesized nanoparticles were sonicated in ultrapure ethanol to obtain stable dispersion. Afterward, one drop of this dispersion was placed on the copper grid with a carbon film.

The average size of nanoparticles (D_TEM_, as mean value) was measured for at least 100 different nanoparticles and at least three different micrographs. In addition, the presence of the modifiers on the surface of nanoparticles was determined using infrared spectroscopy. Fourier-transform infrared (FTIR) spectra were measured for magnetite nanoparticles using the KBr pellet method in infrared transmission mode using a Nicolet 6700/8700 FTIR spectrometer (Thermo Fisher Scientific, Waltham, MA, USA). Magnetic properties were characterized using a vibrating-sample magnetometer LakeShore VSM 7307 at room temperature and in a magnetic field up to 10 kOe. The colloidal stability tests were performed for the water dispersions with 3 mg/ml concentration and 10 mg/ml. For this purpose, the washed and dried magnetite nanoparticles were sonicated with DI water using a UP200St device (26 kHz, 10 W) in a pulse mode. The zeta potential and average size distribution of dispersed particles were analyzed by a Zetasizer Nano ZS (Malvern PANalytical Ltd, United Kingdom) with an autocorrelation function of 10 s and at room temperature. Samples were measured in triplicate. The standard parameters for the characterization of Fe_3_O_4_ (refractive index = 2 and absorbance = 0.01) were chosen.

### Magnetic hyperthermia measurements

The magnetically induced hyperthermia was measured using D5 Series Automatic Driver G2 equipped with a D5 Calorimetry CoilSet device (nanoScale Biomagnetics SL) for water dispersions with a concentration equal to 10 mg/ml. In all measurements, dispersions were sonicated for 10 min just before measuring. Firstly, the role of the magnetite concentration on magnetic hyperthermia was determined for the water dispersions with concentrations equal to 3 mg/ml, 5 mg/ml, and 10 mg/ml. The measurements were performed for a constant frequency equal to 386.5 kHz and magnetic field strength of 27 kA/m. Next, the role of the frequency and magnetic field on the induced hyperthermia was studied for five sets of parameters: 304.7 kHz and 30 kA/m, 347.0 kHz and 26.3 kA/m, 386.5 kHz and 17.1 kA/m, 386.5 kHz and 23.65 kA/m and 386.5 kHz and 27 kA/m. All measurements were repeated three times for the freshly prepared dispersions. The reproducibility of the induced effect by the same dispersion (cyclic-induced hyperthermia) was tested for chosen sample by heating and cooling the same sample at 386.5 kHz and 27 kA/m five times. Finally, the magnetically induced hyperthermia was determined for much lower magnetic fields (5, 10, and 15 kA/m) and constant frequency (304.7 kHz).

### Cytotoxicity assay

Human dermal fibroblasts (PDF1) were purchased from the American Type Cell Culture Collection (ATCC, Manassas, VI, USA). Cells were cultured in Dulbeco's Modified Eagles Medium (DMEM, Sigma Aldrich, Germany) with high glucose (5 g/L) and supplemented with 10% heat-inactivated Fetal Bovine Serum (FBS, PAN Biotech, Germany), antibiotics (1000 U/ml penicillin, 100ug/ml streptomycin, 250 ug/ml amphotericin B (PAA Laboratories GmbH, Austria)) and 2 mM L-Glutamine (PAA Laboratories GmbH, Austria). To ensure the absence of the ethyl acetate, samples were washed with DI water and dried at 60 °C overnight. The stable nanoparticle dispersions with a starting concentration of 0.25 mg/ml in DI water were prepared for the experiments. Cells were cultured in a humidified atmosphere with 5% CO_2_ at 37 °C. When obtaining 90%, confluence cells were passaged to a new 75cm^2^ culture dish and reduced to 1:3. Cells at passage 9 were used for experiments. Cytotoxicity assays were performed in a 48-well plate format. A day before, test cells were seeded at a density of 25·10^3^ cells per well in 250 µL of culture medium. The next day nanoparticles were added to reach a final concentration of 10, 25, 50, 75, and 100 µg/mL and incubated for 24, 48, and 72 h. After this time culture medium was replaced with 120 µL of 10% solution of AlamaBlue (Thermo Scientific, Germany) in the culture medium and incubated for 1 h. After this time, 100 µl were collected, and fluorescence was measured using VICTOR X5 multilabel plate reader (Perkin Elmer, USA) at EM/EX 590/560 nm. Results were presented as a percentage of control (untreated) cells. To visualize cells viability, after 7 2 h of incubation with nanoparticles, the culture medium was replaced with PBS containing 5ug/ml of Ethidium Bromide (Thermo Scientific, Germany) and 0,2 nM of Fluorescein Diacetate (FDA, Sigma Aldrich, Germany), incubated for 10 min and observed under a fluorescent microscope.

### Ultrastructure of PDF1

The ultrastructural changes in fibroblast cells were determined based on TEM analysis. Fibroblasts were treated with NPs at a 50 µg/ml concentration for 24, 48, and 72 h. Then detached by trypsinization, the cell suspension was prepared according to the standard TEM analysis method: washed, dehydrated, and embedded in epoxy resin (Epoxy Embedding Medium Kit; Sigma)^39,40^. Ultra-thin Sects. (70 nm) were cut on a Leica Ultracut UCT25 ultramicrotome and stained with uranyl acetate and lead citrate. The material was analyzed using a Hitachi H500 transmission electron microscope at 75 kV^41^.

## Results and discussion

### Magnetite nanoparticles' structure and morphology

**Figure 1:**
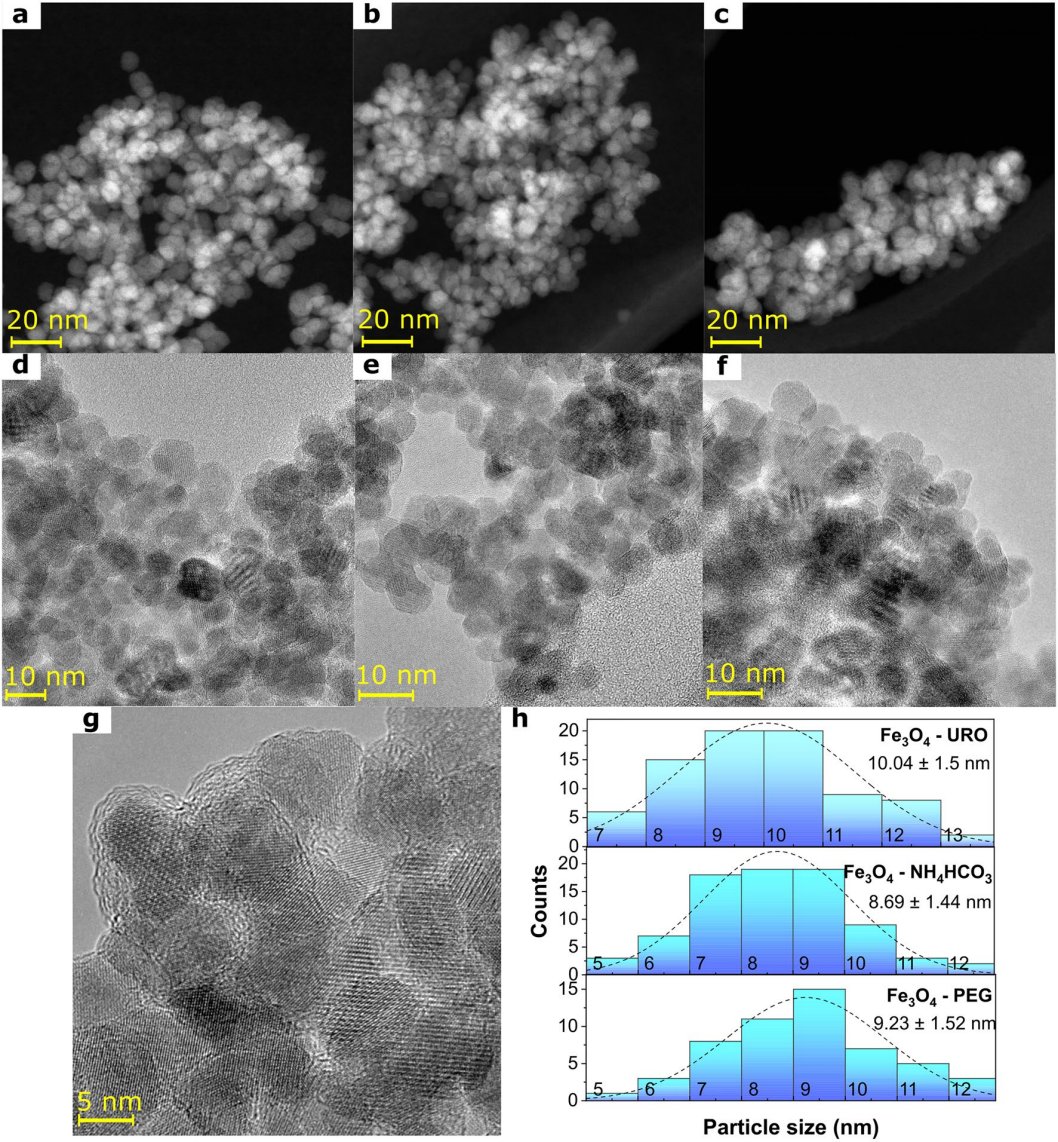
Results of transmission microscopy images analysis obtained for Fe_3_O_4_—URO NPs (**a**, **d**), Fe_3_O_4_—NH_4_HCO_3_ NPs (**b**, **e**) and Fe_3_O_4_—PEG NPs (**c**, **f,** and **g**); (**a**-**c**) high-angle annular dark-field scanning transmission electron microscopy images; (**d**-**g**) transmission electron microscopy images; (**h**) histograms of particle size distribution.

**Figure 2:**
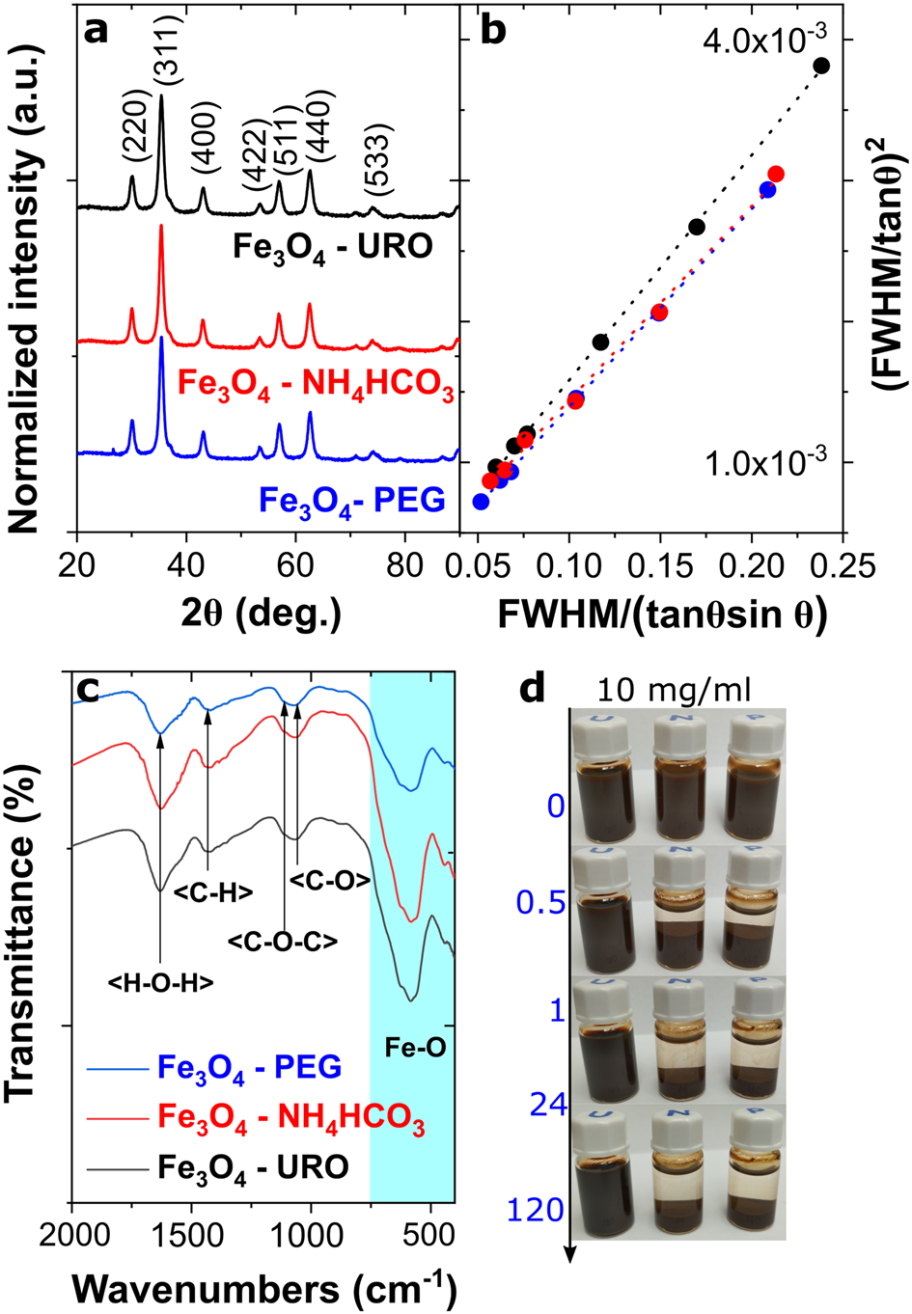
(**a**) XRD patterns of synthesized magnetite nanoparticles with characteristic Miller indices (Fd-3 m space group; 00–019-0629 card number), (**b**) Halder-Wagner plots from (220), (311), (400), (422), (440) and (511) diffraction peaks (R^2^ above 0.99 in all cases), (**c**) FTIR spectra of magnetite nanoparticles with marked identified vibrations related to the presence of the functionalized surface, (**d**) macroscopic images of the changes in the stability of colloidal dispersion of magnetite nanoparticles with high 10 mg/ml concentration in the time domain, i.e. from 0 to 120 h (from left: Fe_3_O_4_—URO NPs, Fe_3_O_4_—NH_4_HCO_3_ NPs and Fe_3_O_4_—PEG NPs).

The structure and morphology of magnetite nanoparticles were studied using X-ray diffraction (XRD) and transmission electron microscopy (TEM). Firstly, the shape and size of magnetite nanoparticles were determined based on the TEM images analysis (Fig. 1a–h). As can be seen, in HAADF STEM (Fig. 1a–c) and TEM (Fig. 1d–f) images, all nanoparticles are characterized by nearly spherical shapes and form agglomerated structures in powder forms. Interestingly, only in the case of Fe_3_O_4_—PEG NPs, the core–shell structure was observed in the TEM image (Fig. 1g), and this nanostructure is related to the visible, nanometric-size polymer coating. Moreover, it can be concluded that this amorphous-like shell is attributed to the presence of PEG on the magnetite core surface because this structure was not visible on TEM images for Fe_3_O_4_—URO and Fe_3_O_4_—NH_4_HCO_3_ NPs. The presence of a typical for magnetite spinel structure was confirmed based on the XRD patterns analysis (Fig. 2a). All identified diffraction peaks were attributed to the magnetite phase (Fd-3 m space group; 00-019-0629 card number). The observed broadening of the peaks is related mainly to nanosized materials' ultrafine crystallite size and internal strain. Accordingly, the average crystallite size (*D*_*XRD*_) was calculated using the Halder-Wagner method and is listed in Table 1. In this method influence of both ultrafine size and strain on the broadening of the diffraction peaks is considered according to the Eqs. (1)–(3)^42–44^.

1 $$\left( {\frac{{\beta^{*} }}{d}} \right)^{2} = \frac{1}{{D_{XRD} }}\frac{{\beta^{*} }}{{d^{2} }} + \left( {\frac{\varepsilon }{2}} \right)^{2}$$

2 $$\beta^{*} = \frac{{\left( {FWHM} \right)cos\theta }}{\lambda }$$

3 $$d = \frac{2sin\theta }{\lambda }$$

where *D*_*XRD*_ is the average crystallite size, *ε* is the microstrain, *FWHM* is the full width at half maximum of the diffraction peak, *θ* is the diffraction angle, and *λ* is the X-ray wavelength. According to that, it is possible to rewrite Eq. (1) and determine *D*_*XRD*_ from the slope of the obtained curve on the Halder-Wagner plots (see Fig. 2b):

4 $$\left( {\frac{FWHM}{{\tan \theta }}} \right)^{2} = \frac{K\lambda }{{D_{XRD} }}\frac{FWHM}{{\tan \theta \sin \theta }} + \left( {\frac{\varepsilon }{2}} \right)^{2}$$

where* K* is a constant equal to 0.94 in the analyzed case of magnetite nanoparticles.

**Table 1:** Comparison of the size of the nanoparticles determined based on the analysis of TEM images (*D*_*TEM*_) and average crystallite size calculated using the Halder-Wagner method (*D*_*XRD*_) together with the values of aggregates diameter measured using DLS technique (*D*_*DLS*_) and zeta potential values (ζ potential) determined for the 10 mg/ml water dispersions.

Sample	D_XRD_ (nm)	D_TEM_ (nm)	D_DLS_ (nm)	ζ potential (mV)
Fe_3_O_4_—URO	9.06	10.04 ± 1.5	120.57 ± 1.99	26.03 ± 0.55
Fe_3_O_4_—NH_4_HCO_3_	10.51	8.69 ± 1.44	332.73 ± 13.14	9.29 ± 1.91
Fe_3_O_4_—PEG	10.31	9.23 ± 1.52	523.77 ± 4.62	14.53 ± 1.02

The influence of the synthesis method on the surface chemistry and colloidal stability in water was additionally tested, and obtained results are presented in Fig. 2c and d. As can be seen, despite using different modifiers, the magnetite surface in all cases was functionalized by the glycol molecules—triethylene for Fe_3_O_4_—URO NPs and Fe_3_O_4_—NH_4_HCO_3_ NPs and PEG on the surface of Fe_3_O_4_—PEG NPs, which is consistent with TEM images analysis. The absence of the N–H vibrations for samples synthesized using urotropine and NH_4_HCO_3_ can be related to the decomposition of both compounds under the synthesis. Formed in this process products, including ammonia, can affect functionalization through interaction with the magnetite surface. However, the NH_4_HCO_3_ decomposition appears at low temperatures, even below the temperature of Fe(acac)_3_ decomposition; therefore, the role of this modifier should be much smaller than in the case of urotropine.

Typical for ultrafine magnetite nanoparticles, Fe–O bonds were observed in the range of 400–650 cm^−1^^12^. The C–O–C ether stretching vibration related to the presence of -CH_2_–OH–CH_2_- in glycols was observed at around 1100 cm^-1^^45^. Also, the characteristics of ethylene glycol-coated magnetite nanoparticle vibrations related to the C-H bending and C–O stretching were observed at around 1429 and 1054 cm^-1^, respectively^35,46^ Broad vibration at around 1633 cm^-1^ corresponds to the H–O–H deformation peak^47,48^. Even though the glycol molecules functionalize all nanoparticles, and their size is similar, their colloidal stability is not the same. The most stable colloids were received from the Fe_3_O_4_—URO NPs, even at a high magnetite concentration of 10 mg/ml. Fe_3_O_4_—NH_4_HCO_3_ NPs and Fe_3_O_4_—PEG NPs sedimented in the dispersion quickly, after 30 min, while Fe_3_O_4_—URO NPs water dispersion without any sedimentation can be stored for five days and longer (Fig. 2d). According to the FTIR analysis, the urotropine does not functionalize the surface of Fe_3_O_4_ but still plays a part in synthesizing nanoparticles with high colloidal stability. It is possible that the degradation products of the urotropine (i.e. ammonia and formaldehyde), which appear above 200 °C, play a part in this process^49^.

According to these unexpected observations, the zeta potential values (ζ potential) and the aggregate diameter (*D*_*DLS*_) were measured using the dynamic light scattering (DLS) method for the water dispersions. The analysis results are presented in Table 1 for the dispersion with a concentration equal to 10 mg/ml. As can be seen, the magnetite nanoparticles synthesized in the presence of urotropine are characterized by the lowest aggregate diameter value, above 4-times lower than Fe_3_O_4_—PEG NPs and above 2.5-times lower than for Fe_3_O_4_—NH_4_HCO_3_ NPs. The same tendency was observed for the lower concentration value (Table S1); however, while the aggregate diameter value for Fe_3_O_4_—URO NPs do not depend on the dispersion concentration, the values of the *D*_*DLS*_ for two other samples are much lower compared to the 10 mg/ml dispersions. The ζ potential of all nanoparticles is positive, characteristic of the coated magnetite nanoparticles^35^. Generally, nanoparticles with a zeta potential less than -25 mV and higher than + 25 mV are characterized by high colloidal stability, which confirms the observations noted for the Fe_3_O_4_—URO dispersions. The Fe_3_O_4_—NH_4_HCO_3_ and Fe_3_O_4_—PEG have much lower ζ potential values, equal to 9.29 ± 1.91 and 14.53 ± 1.02 mV, which result in the formation of much bigger aggregates and formation of unstable dispersions.

The zeta potential values differ for low (3 mg/ml; see Table S1) and high (10 mg/ml) concentrations. However, the similar ζ potential changes between Fe_3_O_4_—URO, Fe_3_O_4_—PEG and Fe_3_O_4_—NH_4_HCO_3_ can be easily observed also for low concentration. These changes in zeta potential values are typical and discussed before by Kaszuba et al.^50^. According to their studies, obtained herein values (for high concentrations) must be treated as the relative zeta potential values, not absolute ones. However, it is more relevant than the absolute one because it describes the dispersions' properties, with a concentration equal to this one, which will be tested further in the magnetic hyperthermia measurements.

### Magnetic properties

**Figure 3:**
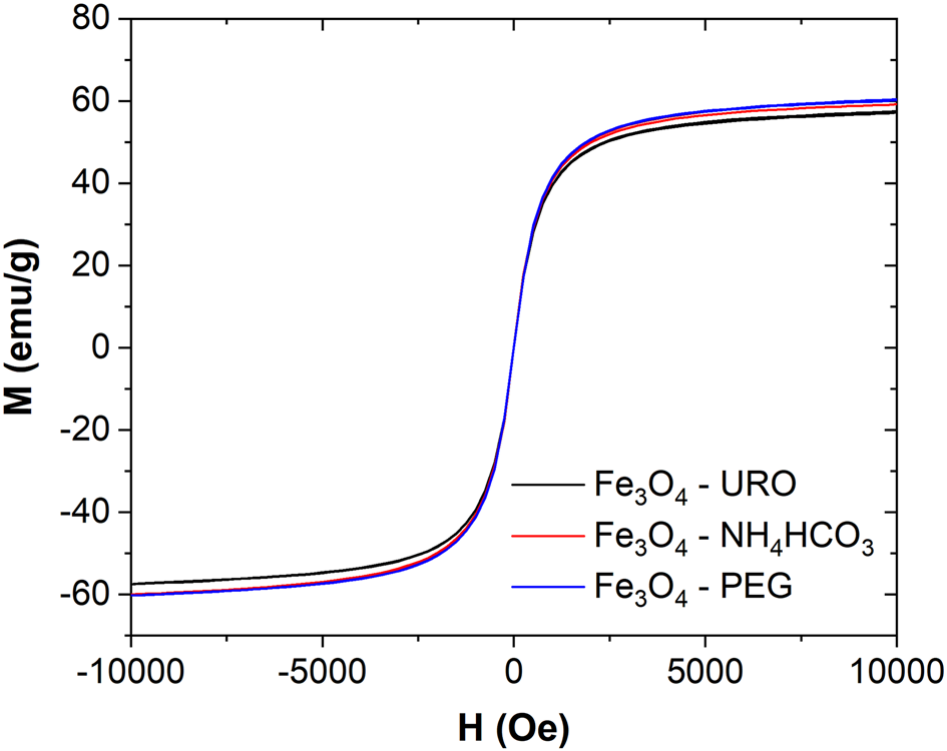
Hysteresis loops of the superparamagnetic magnetite nanoparticles synthesized in the presence of urotropine (Fe_3_O_4_—URO NPs), NH_4_HCO_3_ (Fe_3_O_4_—NH_4_HCO_3_ NPs) and polyethylene glycol (Fe_3_O_4_—PEG NPs).

The nanoparticles, which can be used in magnetic hyperthermia applications, are mostly single-domain particles with superparamagnetic or ferromagnetic properties. Generally, the ferromagnetic properties, according to the larger hysteresis losses, generate more heat under alternating magnetic fields^51^. On the other hand, in the case of the superparamagnetic particles, while the external magnetic field is removed, the nanoparticles are completely unmagnetized, which results in their unique advantage for biomedical applications (not only as magnetic hyperthermia agents but also as drug delivery systems)^52,53^. Accordingly, the magnetic properties of synthesized nanoparticles were determined based on the VSM curves (Fig. 3). As can be seen, despite the different organic modifiers, all synthesized samples are characterized by a superparamagnetic state. Only slight differences between saturation magnetization (*M*_*s*_) can be observed. The highest value of *M*_*s*_ have Fe_3_O_4_—PEG and Fe_3_O_4_—NH_4_HCO_3_ NPs (60.4 and 60.1 emu/g), while Fe_3_O_4_—URO NPs have *M*_*s*_ equal to 57.5 emu/g. The changes in coercivity (*H*_*c*_) are also slight, and the value of this parameter is around 1 Oe, which confirms the superparamagnetic state of all magnetite nanoparticles.

### Magnetic hyperthermia

Magnetic hyperthermia is one of the most promising cancer treatments, which is related to the possibility of using biocompatible magnetic nanoparticles such as magnetite and treatment of the cancer cells only locally without any significant negative impact on the healthy cells. Unfortunately, there is no one simple rule of the maximum temperature to which the tumour cells should be heated without impact on the healthy cells. Generally, this temperature should be in the range of 39–45 °C, above which thermal ablation of cells can occur^24^. In the case of the magnetic nanoparticles, the heat generated by them is related to Neel relaxation and Brownian relaxation. The first process relates to the reorientation of magnetic moments parallel to a magnetic field, while the second relates to the movement of whole nanoparticles in an external magnetic field^54^. Accordingly, the value of the generated thermal energy can be easily modified by synthesizing nanoparticles with different sizes, shapes, agglomeration ratios and even core–shell nanostructures and by the changes in alternating magnetic field (AMF) frequency and strength^55^.

In the case of the magnetic-induced hyperthermia effect, various parameters influence the generated effect. Accordingly, this study determined the influence of magnetite nanoparticle concentration, AMF frequency (*f*) and strength (*H*) for Fe_3_O_4_—URO NPs, Fe_3_O_4_—NH_4_HCO_3_ NPs and Fe_3_O_4_—PEG NPs. Firstly, nanoparticle concentration influence on the magnetically indued hyperthermia was determined for three different concentrations, constant frequency, and magnetic field strength. Next, the influence of the frequency and strength was measured for high *H·f* parameters in the range of 6.6 ∙10^9^ A/ms to 10.4 ∙10^9^ A/ms following the newly developed application criteria, and the best sample was chosen to study specific absorption rate and intrinsic loss power parameters. The SAR (Specific Absorption Rate) determine the capacity of a magnetic material to absorb energy from an alternating magnetic field and is defined as the amount of power absorbed by the sample per mass unit. In the case of the studied magnetic colloids, this power can be expressed as the amount of energy converted into heat per time and mass. Accordingly, the SAR value can be calculated using Eq. (5).

5 $$SAR = \frac{1}{{m_{np} }}\frac{Q}{\Delta t}$$

where *Q* is the generated heat, *m*_*np*_ is the mass of the nanoparticles, and *Δt* is the time in which heat was generated. The generated heat in the used adiabatic system can be determined based on the calorimetric approach and expressed as:

6 $$Q = \left( {m_{np} c_{np} + m_{l} c_{l} } \right)\Delta T$$

where *c*_*np*_ and *c*_*l*_ are the specific heat capacity of the nanoparticles and liquid carrier and *m*_*np*_ and *m*_*l*_ mass of the nanoparticles and liquid carrier, *ΔT* is the change in the temperature generated by the AMF. Assuming that the *m*_*np*_*c*_*np*_ <  < *m*_*l*_*c*_*l*_ and the colloid concentration of sample *C* is equal to *m*_*np*_*/V*_*l*_ the SAR can be expressed as:

7 $$SAR = \frac{{\delta_{l} c_{l} }}{C}\left( {\frac{\partial T}{{\partial t}}} \right)_{max}$$

where *δ*_*l*_ is the liquid carrier density, and $\left( {\frac{\partial T}{{\partial t}}} \right)_{max}$ is the maximum heating rate of the colloid approximated by the modified Box-Lucas model (MBL):

8 $$\left( {\frac{dT}{{dt}}} \right)_{max} = A \cdot B = \left( {T_{eq} - T_{0} } \right)\frac{1}{\tau }$$

where *T*_*eq*_ is the equilibrium temperature of the colloid, *T*_*0*_ is the initial temperature of the colloid, and *τ* is the characteristic heating time depending on sample properties. Accordingly, the SAR value can be expressed as:

9 $$SAR = \frac{{\delta_{l} c_{l} }}{C}\left( {A \cdot B} \right)$$

While the SAR depends on the magnetic field strength and frequency, the intrinsic loss power (ILP; Eq. 10) parameter was introduced to compare the laboratory results. However, the applicability of this parameter is also limited (dispersity of the sample must be higher than 0.1, the magnetic field must be below the saturation, the frequency must be in the range of 10^5^ and 10^6^ and also thermodynamic losses should be lower or equal to power input)^56^. Furthermore, this parameter assumes the quadratic dependence with the magnetic field (*H*) and linear dependency with frequency (*f*) of the SAR parameter and is correct, while linear response theory can be applied^57^.

10 $$ILP = \frac{SAR}{{fH^{2} }}$$

Various models were proposed in the literature to describe magnetically induced hyperthermia, including linear response theory (LRT), Stoner–Wohlfarth model and equilibrium functions^58^. For the superparamagnetic nanoparticles approximation of heat generated by them was proposed as LRT by Rosensweig^59^, in which dissipated power (P) can be represented as:

11 $$P = \pi \mu_{0} \chi^{^{\prime\prime}} H^{2} f$$

where *µ*_*0*_ is the permeability of free space, *χ"* is the imaginary part of complex susceptibility, *H* is the magnetic field strength, and *f* is the magnetic field frequency. Various factors restrict the applicability of this model, while the most important one is small magnetic fields, for which the assumption is that the magnetization changes linearly with increasing the magnetic field can be applied^60^. Accordingly, the possible applicability of LRT in the studied magnetite nanoparticles was first verified. As can be seen in Fig. S1, two regions can be observed. While the magnetic field is equal to or lower than 17.1 kA/m, the LRT can be applied to describe the behaviour of synthesized superparamagnetic nanoparticles. Above that field value, this model cannot be applied, which is consistent with the literature data^60^. This LRT region obeys results obtained for all synthesized samples and *H·f* product from 1.52 to 6.61·10^9^ A/ms. Therefore obtained ILP values (calculated only for this region) can be compared with other literature data, for which LRT also can be successfully applied to describe magnetically induced hyperthermia.

The influence of the magnetite nanoparticle concentration on the SAR value is nonlinear^61,62^. Accordingly, three concentrations were measured to determine the optimum concentration for further characterization. The typical non-monotonic behaviour was observed (see Fig. S2). While the highest SAR values were observed for the 10 mg/ml concentration, the lowest SAR values were obtained for all 5 mg/ml magnetite nanoparticles colloids, despite the differences between their colloidal stability and aggregate size. The presented behaviour is typical for the transition between the single-particle scenario (observed for 3 mg/ml dispersions) and the collective-particle scenario observed and described by Conde-Leboran et al.^61,62^.

**Figure 4:**
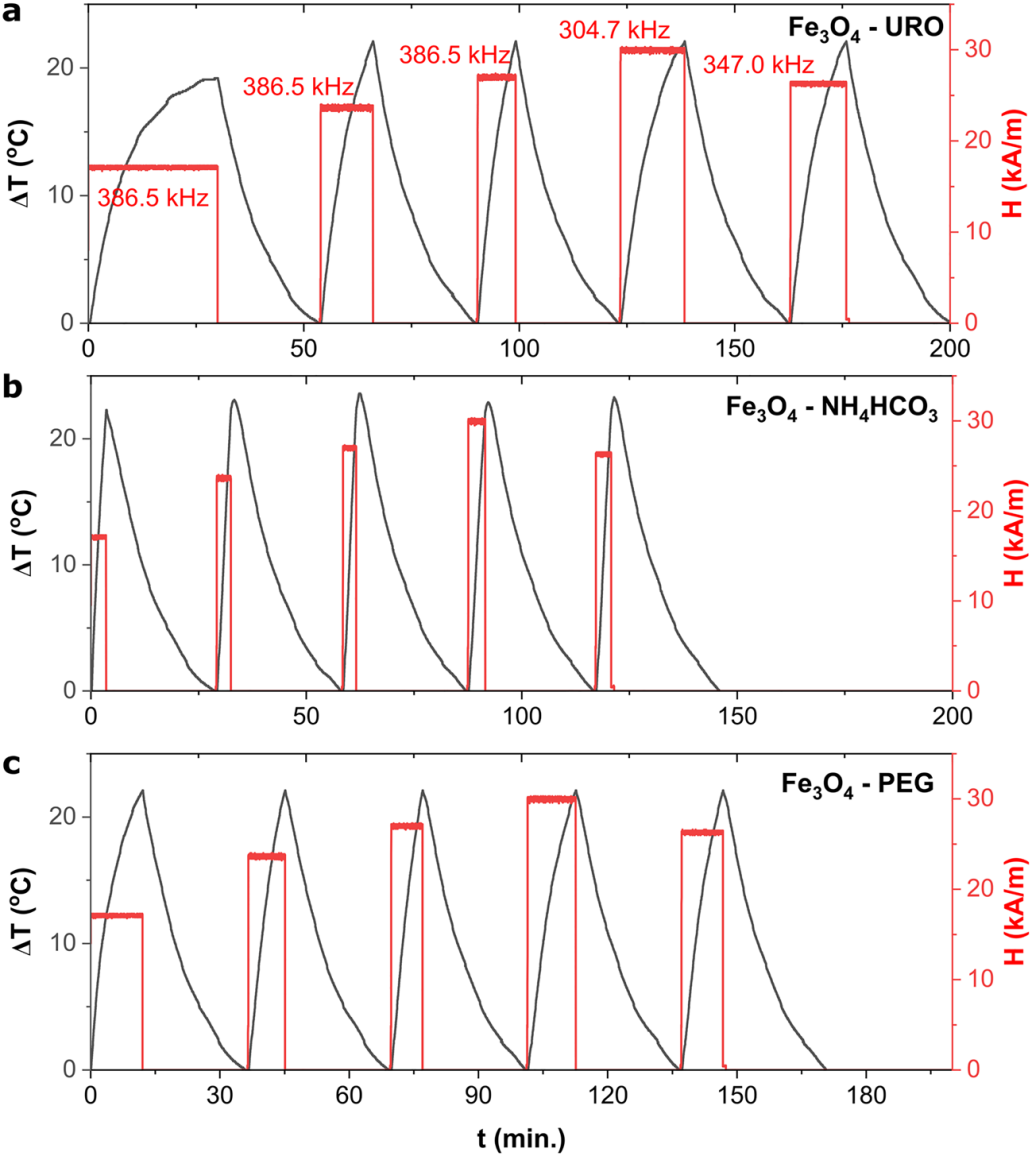
Influence of magnetic field (frequency and strength) on the temperature changes induced by magnetite nanoparticles dispersions with a concentration of 10 mg/ml measured for Fe_3_O_4_—URO NPs (**a**), Fe_3_O_4_—NH_4_HCO_3_ NPs (**b**) and Fe_3_O_4_—PEG NPs (**c**).

According to the above, the 10 mg/ml concentration was chosen for further analysis, for which a relatively high magnetically induced hyperthermia effect is related to the formation of collective particles. The frequency and magnetic field strength analysis on the SAR and ILP parameters are presented in Fig. 4 and Table 2. As can be seen, the temperature changes generated by the external AMF are different for all samples. Only Fe_3_O_4_—NH_4_HCO_3_ NPs are characterized by ultrafast temperature growth in all tested frequencies and magnetic field strength. This can be related to the relatively high aggregate diameter (332.73 ± 13.14 nm) and lowest zeta potential value equal to 9.29 ± 1.91 mV, which reflects the higher tendency to form aggregates. For other samples, a slow temperature increase can be observed, especially using 386. 5 kHz and 17.1 kA/m. What is essential, for the Fe_3_O_4_—NH_4_HCO_3_ NPs also, the highest SAR (69.6 ± 5.2 W/g) and ILP (0.613 ± 0.051 nHm^2^/kg) values observed for the lowest tested *H·f* parameter equal to only 6.6 ∙10^9^ A/ms, while for example, for the Fe_3_O_4_—URO NPs the highest SAR was equal only to 38.8 ± 1.3 W/g for the *H·f* equal to 10.4 ∙10^9^ A/ms. Moreover, calculated SAR and ILP values are the highest for nanoparticles synthesized in the presence of NH_4_HCO_3_ in all analyzed frequency and magnetic field ranges. This phenomenon can be related to collective behavior (aggregation of ultrafine nanoparticles). However, the changes between magnetic hyperthermia behavior and aggregate size are complex, and various (sometimes opposites) models were proposed^63^.

**Table 2:** Specific absorption rate (SAR) and intrinsic loss power (ILP) calculated for magnetite nanoparticles dispersions (10 mg/ml).

Frequency (kHz)	Field intensity (kA/m)	SAR (W/g)	ILP (nHm^2^/kg)
Fe_3_O_4_—URO NPs			
386.5	17.1	13.4 ± 2.0	0.119 ± 0.018
386.5	23.65	31.8 ± 0.5	n.a
386.5	27	38.8 ± 1.3	n.a
304.7	30	28.2 ± 1.5	n.a
347.0	26.3	30.2 ± 2.2	n.a
Fe_3_O_4_—NH_4_HCO_3_ NPs			
386.5	17.1	69.6 ± 5.2	0.613 ± 0.051
386.5	23.65	48.4 ± 3.0	n.a
386.5	27	52.7 ± 2.9	n.a
304.7	30	42.6 ± 3.7	n.a
347.0	26.3	45.1 ± 1.9	n.a
Fe_3_O_4_—PEG NPs			
386.5	17.1	25.8 ± 2.8	0.228 ± 0.025
386.5	23.65	36.8 ± 1.7	n.a
386.5	27	42.7 ± 2.9	n.a
304.7	30	35.0 ± 1.7	n.a
347.0	26.3	38.4 ± 0.2	n.a

Moreover, the changes in aggregate size and even shape (formation of the chains under applied magnetic field) also affect the SAR value^13^. The theoretical modeling performed by Abu-Bakr et al.^64^ shows that the clusterization of nanoparticles decreases the thermal effect. Similar findings were presented in^65^ and can be used to describe changes between SAR values of Fe_3_O_4_—NH_4_HCO_3_ NPs and Fe_3_O_4_—PEG NPs (nanoparticles synthesized in presence of PEG have a higher aggregate diameter, therefore, have lower SAR values in the analyzed frequency and field strength range). In the case of the sample synthesized in the presence of urotropine, it is expected that according to the high colloidal stability and the smallest aggregate size, it should have the highest SAR values, which were not observed herein. This behavior can be related to the changes in the magnetic behavior of the ferrofluid. As mentioned previously, an analyzed concentration equal to 10 mg/ml resulted in appearing of a collective-particle scenario. Therefore, observed changes in SAR values are related to the heat generated not by a single superparamagnetic nanoparticle but by aggregates. Although, as seen in Fig. S2, the changes generated by the aggregate size are not linear, the size of Fe_3_O_4_—NH_4_HCO_3_ NPs aggregates is probably optimal, in which magnetic hyperthermia effect in the collective-particle scenario is the highest. The mechanism responsible for that is still unknown; however, it can be related to the evolution of aggregate size and shape under measurement^13^.

**Figure 5:**
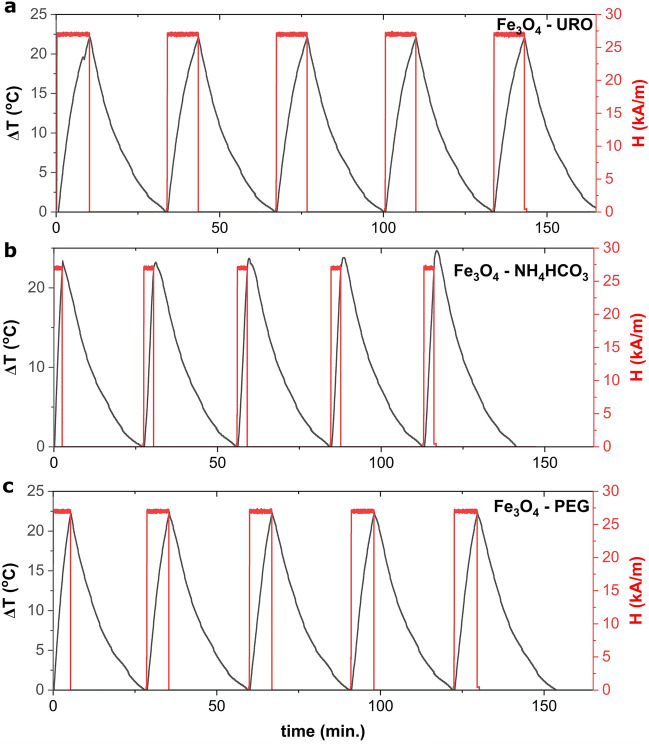
Repeatability of hyperthermia induced by the same sample of magnetite nanoparticles with the concentration of 10 mg/ml and in the constant alternative magnetic field parameters (386.5 kHz, 27 kA/m).

The reproducibility of the same magnetofluid in the case of cyclic magnetically induced hyperthermia was checked and presented in Fig. 5. As can be seen, the sample characterized by the highest SAR value (Fe_3_O_4_—NH_4_HCO_3_ NPs) cannot be used for cyclic magnetically induced hyperthermia; the SAR decreases from 93.17 W/g to 57.06 W/g. Interestingly, the highest decreases were observed in the two first runs (Δ SAR equal to 17.29 W/g); afterwards, SAR stabilized at about 60 W/g. Moreover, the changes induced by the magnetic field change from exponential to linear behaviour. Interestingly, in the case of the Fe_3_O_4_—PEG NPs, the changes are not so visible, and SAR remain at a similar level (for example, 48.97 W/g for the first and 47.04 for the fourth run); also, temperature changes has typical exponential behaviour in the whole experiment. The lowest changes were observed for the Fe_3_O_4_—URO NPs, for which SAR remained at the same level throughout the experiment. i.e. average SAR is equal to 33.20 ± 1.15 W/g. It can be noted that when ζ potential decreases from 26.03 ± 0.55 to 9.29 ± 1.91 mV, the SAR stability also decreases. This behavior can be related to the agglomeration of nanoparticles under external magnetic fields. After the stable agglomerates' formation, the SAR value remains similar.

If we look at the potential application of this cyclic-induced hyperthermia effect, one of the most important use is controlled drug release therapy. For example, magnetite nanoparticles can be coated by thermoresponsive polymers to ensure that the drug is released only when the temperature rises. Ferjaoui et el.^4^ have shown that the synthesis of the core/shell magnetite-based nanoparticles with doxorubicin-loaded thermoresponsive copolymer shell composed of 2-(2-methoxy)ethyl methacrylate and oligo(ethylene glycol)methacrylate results in the formation of the nanostructure, in which drug release with higher yield can be observed when the temperature raises. The possibility of the controlled release of small doses of anticancer drugs supported by the magnetically induced hyperthermia for a long time only close to the cancer cells should be another approach to decrease the toxicity of the anticancer drugs on healthy cells. Accordingly, stable and repeatable heating properties should be required; therefore, Fe_3_O_4_—URO NPs with the lowest ΔSAR value would be a better choice than Fe_3_O_4_—NH_4_HCO_3_ NPs characterized by the highest SAR values.

One of the most critical parameters influencing magnetic hyperthermia is magnetic field frequency and strength. The first commercially developed equipment to treat human patients works at a frequency of 100 kHz with a magnetic field strength equal to 18 kA/m^66^. In the case of the frequency choice, many studies mentioned which is safe for the body ranges. For example, Khan et al. proposed clinical trials ranging from 50 kHz to 2 MHz to avoid skeletal muscle stimulations and magnetic field penetration to the depth tissue^67^. Other research limited the frequency range to 0.1 MHz, above which the peripheral nerve excitation threshold can be significantly raised. To choose the magnetic field strength, the Atkinson-Brezovich criterion or modified version proposed by Hergt and Dutz should be applied^68,69^. According to the modified version, the product between frequency and magnetic field strength should be lower than 5∙10^9^ A/ms when the magnetic hyperthermia is limited to the small body part. However, while the Atkinson-Brezovich criterion was introduced based on the testing of the discomfort of the person treated using a loop with a diameter of about 30 cm, the others were not experimentally tested, especially on the functions of cells^60^. Considering that the novel magnetically induced hyperthermia is based on nanoparticles, not micro-sized implants, these criteria should be revised one more time. For example, Bellizzi et al.^70,71^ showed that the new criterion could be even two orders of magnitude larger than proposed by Atkinson-Brezovich. Moreover, there are various ways to extend this limit and factors which influence the negative impact of AMF on healthy tissue, such as coil type or use of intermittent AMF^72^.

**Figure 6:**
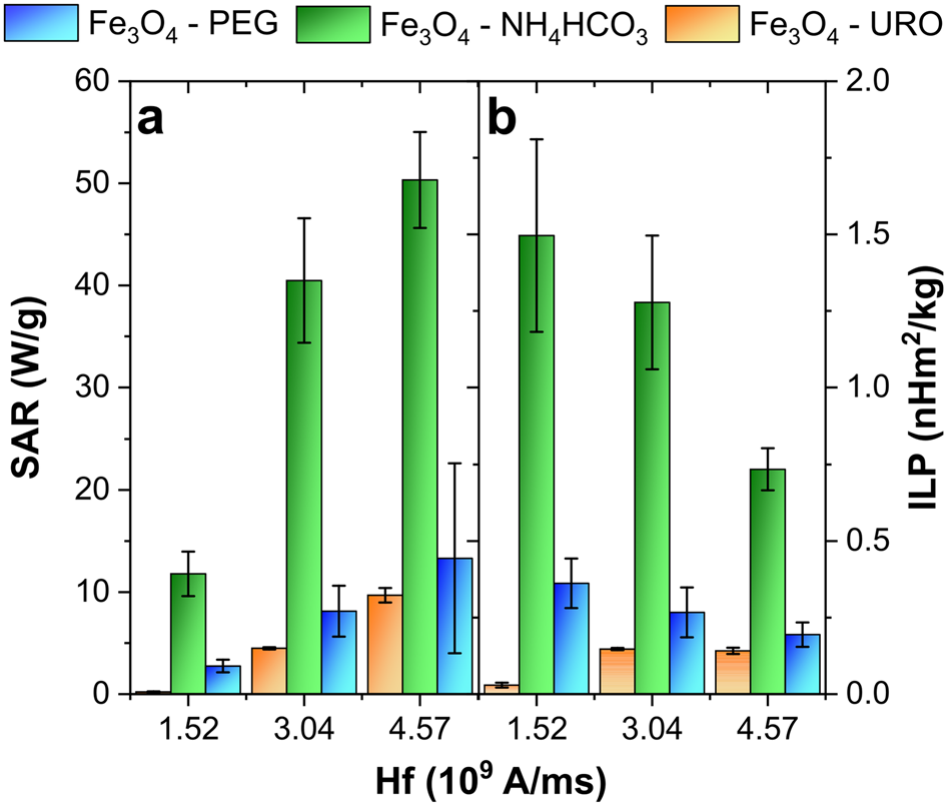
Influence of the *H·f* value on the SAR and ILP parameters determined for Fe_3_O_4_-URO, Fe_3_O_4_- NH_4_HCO_3_ and Fe_3_O_4_-PEG NPs water dispersion with a concentration of 10 mg/ml (for constant field frequency equal to 304.7 kHz).

Accordingly, the influence of the magnetic field on the hyperthermia effect was measured for a fixed frequency equal to 304.7 kHz and three various magnetic fields to comply with the mentioned application criteria. The analysis results are presented in Fig. 6. As can be seen, the differences between tested nanoparticles are visible in the case of both ILP and SAR analysis. The highest SAR and ILP values were received for nanoparticles synthesized in the presence of NH_4_HCO_3_, while the lowest ones characterized Fe_3_O_4_-URO. While the *H·f* product increases from 1.52·10^9^ A/ms to 4.57·10^9^ A/ms, the SAR value increases in all cases; however, the same tendency for ILP was observed only for Fe_3_O_4_-URO. In the case of Fe_3_O_4_- NH_4_HCO_3_ and Fe_3_O_4_-PEG NPS with increasing *H·f* product, the ILP decreases. Therefore, despite the high dispersion stability, the Fe_3_O_4_-URO NPs are characterized by ultralow magnetically induced hyperthermia effect and can be used with higher *H·f* values or for other applications such as drug delivery systems or multifunctional platforms, which dose drugs from the thermoresponsive polymers. In applying nanoparticles for the pure magnetically induced hyperthermia, the Fe_3_O_4_- NH_4_HCO_3_ should be chosen according to the high SAR and ILP values for all *H·f* products (also, higher ones discussed previously in this work).

The applicability of magnetite and ferrites nanoparticles in magnetically induced hyperthermia treatment is widely studied. For similar *H·f* values, it can be seen that higher SAR values have the Fe_3_O_4_- NH_4_HCO_3_ NPs than, for example, tetragonal Mg_0.1_Zn_0.7_Co_0.2_Fe_2_O_4_ and Mg_0.15_Zn_0.65_Co_0.2_Fe_2_O_4_ NPs, for which SAR was around 3.5 and 7.0 W/g for 3.2·10^9^ A/ms. However, obtained results are nearly two times lower than for Mg_0.5_Zn_0.3_Co_0.2_Fe_2_O_4_ NPs, for which SAR was equal to 82.7 W/g^73^. A more interesting comparison can be made based on the data reported by Kullumadil et al.^57^, which introduced the ILP parameter and calculated its value for various commercially available colloids dedicated to magnetic hyperthermia. The tested by them colloids had higher concentrations even above 50 mg/ml; however, obtained herein ILP values for low H-region, in which LRT can be applied, are still higher than, for example, commercially available BNF-02008, BNF-01708, and BNF-01808 (Micromod) with a concentration of 50, 80 and 90 mg/ml, respectively (for both Fe_3_O_4_—PEG_3_ NPs and Fe_3_O_4_—NH_4_HCO_3_ NPs). For the Fe_3_O_4_–NH_4_HCO_3_ NPs, SAR and ILP values were much higher for lower *H·f* product (equal to 1.52·10^9^ A/ms) and equal to 11.8 ± 2.2 W/g and 1.496 ± 0.314 nHm^2^/kg.

### Magnetite nanoparticles cytotoxicity

**Figure 7:**
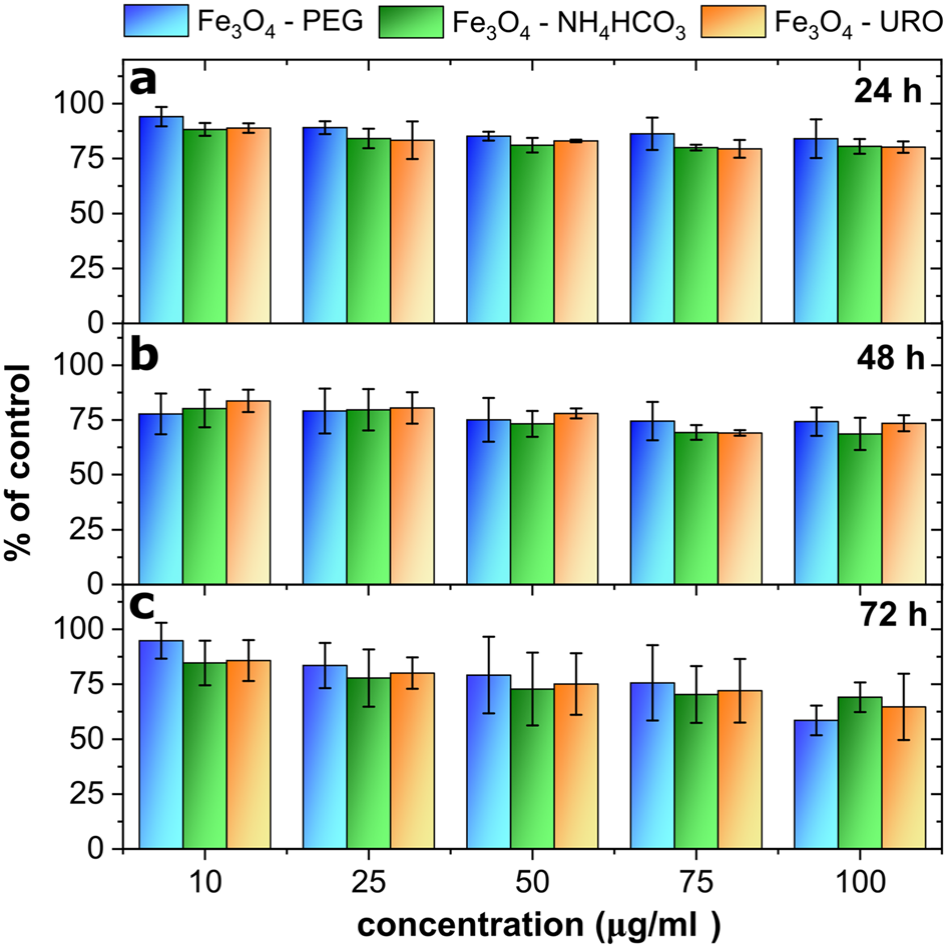
Cytotoxicity of nanoparticles (Fe_3_O_4_—URO NPs, Fe_3_O_4_—NH_4_HCO_3_ NPs and Fe_3_O_4_—PEG NPs) to dermal fibroblasts determined after (**a**) 24 h, (**b**) 48 h and (**c**) 72 h for wide concentration range from 10 to 100 µg/ml.

**Figure 8:**
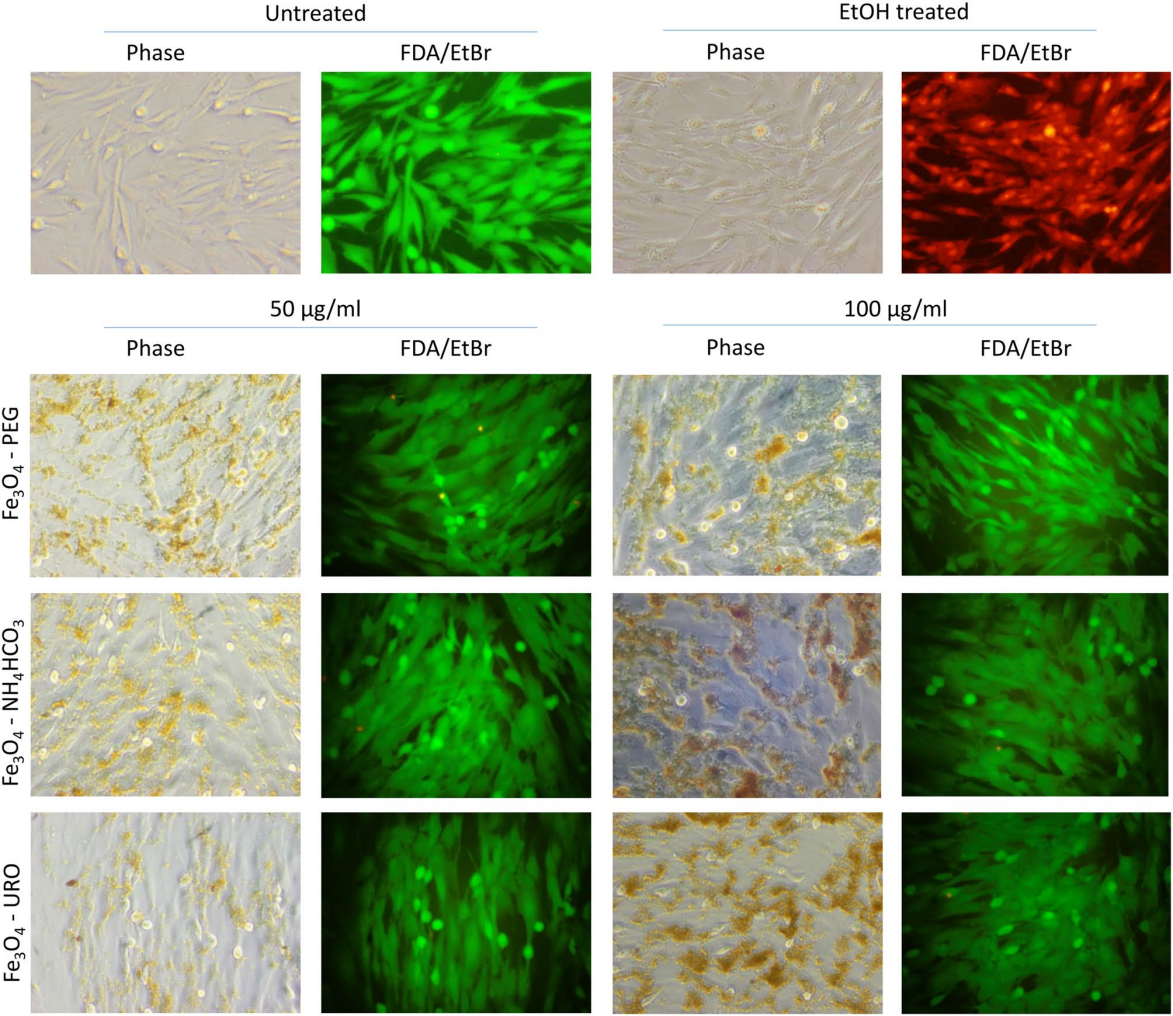
The viability of cells treated with nanoparticles visualized by FDA/EtBr staining after 72 h of culture in the presence of NPs. Cells treated with nanoparticles appear to be alive (green fluorescence). Cells treated with 70% EtOH as a positive control for dead cells characterized by red fluorescence.

Human fibroblasts are used as model cells to study various biological processes because they are involved in the regeneration of tissue damage. Therefore, they can be used as an indicator of the cytotoxicity of nanoparticles used in medicine^74,75^. The first toxicity assay, performed after 24 h of cells incubation with nanoparticles, showed only a slight decrease in cells viability in all tested NPs concentrations (Fig. 7). No differences were observed between the NPs modification. Microscopy analysis of the cell culture revealed that all introduced NPs precipitated from the culture medium and settled down on the cell layer (Fig. S3). Similar data were obtained after 48 h of incubation with NPs, where cells viability decreased only slightly. A more significant decrease in AlamarBlue reduction was detected after 72 h incubation with NPs. The decrease was dose-dependent and most significant at the highest tested concentration of NPs, but cells viability did not drop below 50% of control–untreated cells (Fig. 7). There are no differences in toxicity between types of Fe_3_O_4_ NPs tested for toxicity to dermal fibroblasts at any time. To visualize cells viability by fluorescent microscopy, staining with Ethidium bromide (dead cells—red fluorescence) and Fluorescein Diacetate (live cells—green fluorescence) were performed at 72 h. All treated cells appeared as viable (green fluorescence) (Fig. 8).

**Figure 9:**
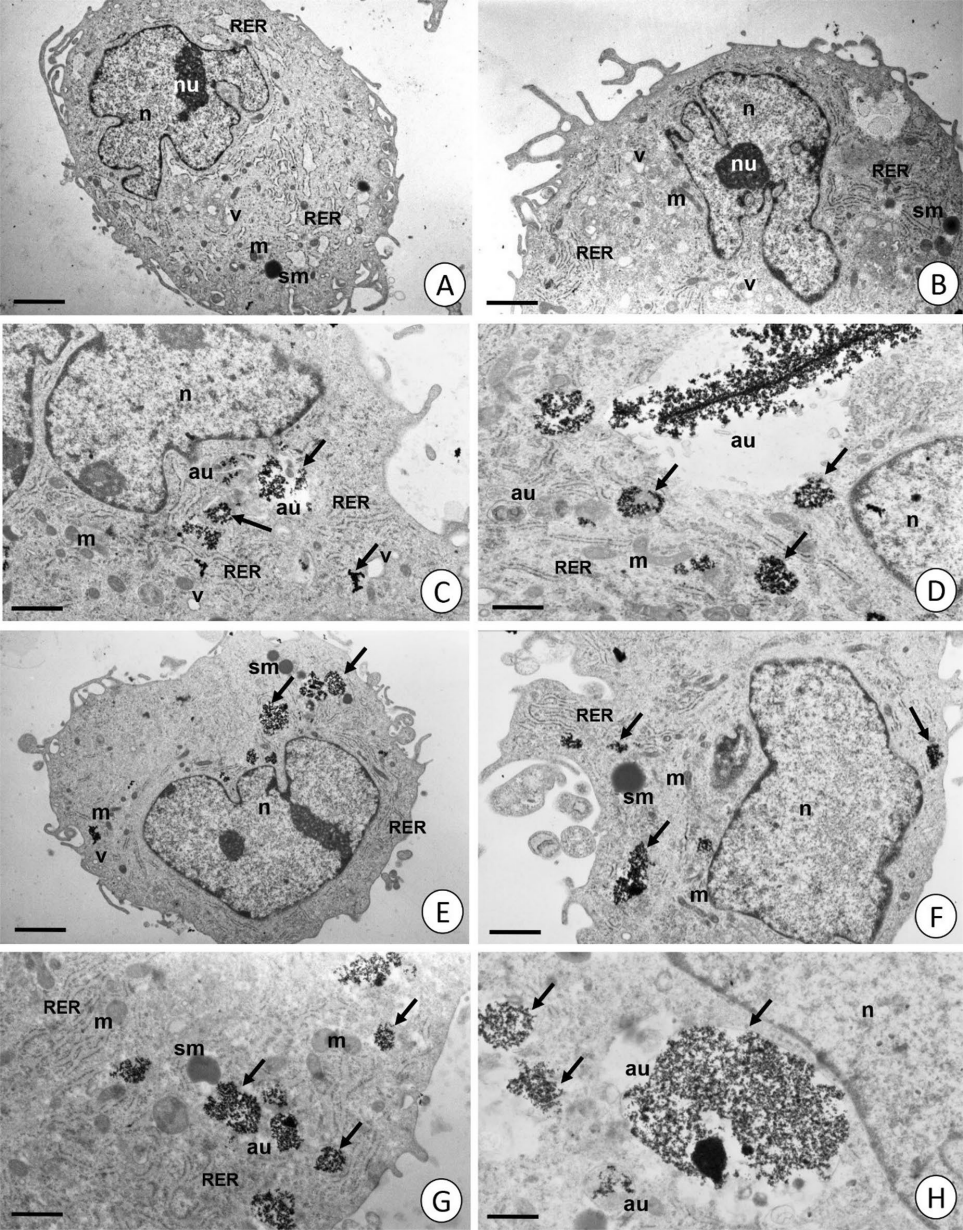
Fibroblasts visible in TEM. (**A**, **B**) 0 – 72 h control group. (**C**, **D**) Fe_3_O_4_—PEG – 72 h experimental group. (**E**, **F**) Fe_3_O_4_—NH_4_HCO_3_ – 72 h experimental group. (**G**, **H**) Fe_3_O_4_—URO – 72 h experimental group. Nuclei (n), mitochondria (m), cisterns of RER (RER), storage material (sm), vacuoles (v), autophagosomes (au), electron-dense granules (arrows). (**A**) Scale bar = 2,37 μm. (**B**) Scale bar = 1,60 μm. (**C**) Scale bar = 1,05 μm. (**D**) Scale bar = 0,73 μm. (**E**) Scale bar = 1,16 μm. (**F**) Scale bar = 1,18 μm. (**G**) Scale bar = 0,92 μm. (**H**) Scale bar = 0,81 μm.

Ultrastructural analysis of NP-treated cells was performed to test whether NPs entered into the cells. Control, untreated cells (marked as 0 group), at each time point (24, 48, and 72 h of the experiment) remained unchanged. The ultrastructure of these cells was taken as the reference for each experimental group at indicated time points (Fig. 9A, B). Numerous flocculent-granular patches of electron-dense material (magnetite nanoparticles) were detected in all experimental groups and time points (Figs. 9C–H, S4C–H, and S5C–H). The material accumulated in the membranous vesicles resembled autophagosomes (Fig. 9C, D, G, H). This material was also observed near the outer surface of the fibroblasts, adhering to their cell membrane. These granules were surrounded by fibroblast cytoplasmic projections and entered their cytoplasm by phagocytosis. The amount of electron-dense material was proportional to the incubation time. Analysis of the cytoplasmic structures of the treated cells in all experimental groups revealed a gradual increase in the number of autophagous structures (autophagosomes, autolysosomes, and residual bodies). Apart from the autophagous structures mentioned, there were no other significant changes in the ultrastructure of treated cells.

Tested nanoparticle seems to be non-toxic to cells in vitro with a shorter incubation time, and only a little toxic effect appears at high doses of NPs after 72 h (Fig. 7), but since all tested Fe_3_O_4_ NPs precipitated (Fig. S3), these results require depth discussion. When NPs settled down, the local concentration of NPs at the bottom of the culture dish increased, and the cells there experienced a much higher concentration of NPs, as assumed in the experiment, suggesting even lower toxicity of tested NPs. On the other hand, NPs precipitation can limit their interaction with cells. We do not know whether precipitation is due to NPs aggregation and precipitation or interaction with cell culture medium compounds. The culture medium contains many organic and inorganic compounds such as ions, glucose, aminoacids, and proteins. It is known that ion presence can affect the NPs aggregation^76^. Also, serum proteins in the culture medium, especially albumins, can interact with NPs creating protein corona around NPs. The presence of protein corona has a rather positive effect, as it prevents NPs from direct interaction with cell membrane decreasing their toxicity^77^. As all tested Fe_3_O_4_ NPs precipitated, any introduced surface modification did not prevent them from interacting with culture medium compounds. As precipitation limits the interaction of NPs with cells and may be the reason for low toxicity, we can indeed conclude from ultrastructural analysis that NPs interact with the cell surface and enter cells by endocytosis. This can suggest that despite NPs precipitation, cells experience NPs effect, which may confirm the reliability of the results. Since these tests were performed in vitro, and many compounds present in the culture medium are also found in body fluids such as blood or lymph, we can predict that in vivo toxicity of Fe_3_O_4_ NPs appears to be low, but further studies should verify this.

Interaction of NPs with the cell membrane can lead to its destruction and cause rapid, necrotic cell death. As revealed by ultrastructural analysis, NPs are in contact with cells' surfaces. The observed toxicity appeared slowly, and we did not observe an accumulation of dead cells in the treated culture; thus, necrosis was excluded. The primary mechanism of toxicity reduction by PEG coating is preventing plasma membrane disruption^28^; thus, the low toxicity of Fe_3_O_4_—PEG NPs may be due to the presence of nanometric polymer coating (see Fig. 1g). As all modified NPs have the same toxicity level, this suggests that NH_4_HCO_3_ and URO modification work as good as PEG in toxicity reduction.

Because toxicity appears only after a longer incubation time at high doses of NPs, it can result from damage accumulation caused by NPs inside the cells. Iron nanoparticles, in proper condition, can cause a certain type of programmed cell death called ferroptosis^78^. Since FDA/EtBr dual staining showed no increase in the number of dead cells (Fig. 8), the decrease in AlamarBlue reduction has other reasons than cell death. Ultrastructural analysis revealed strong induction of the autophagic process in treated cells (Figs. 9, S4, and S5). Autophagy is a physiological process where damaged organelles are degraded, enabling cell regeneration. An increase in the autophagy process is observed in response to hunger or stress.

Induction of autophagy in cells is reported for many types of NPs. It is postulated that autophagy plays a dual role in NPs toxicity. Negative, causing toxicity as intense autophagy can lead to autophagic cell dead and protection by sequestering NPs in autophagosomes and preventing them from interacting with other organelles inside the cell^79,80^. Nevertheless, the autophagic process alters the metabolic process in cells^81^. Thus, decreased alamar reduction may result from intensive autophagy in NPs treated cells. Intensive autophagy inhibits cell growth and proliferation; therefore, lower resazurin reduction can be an effect of decreased cell proliferation. This is more likely as the difference in cells proliferation needs more time to be visible in the metabolic assay. Since the exact mechanism of autophagy induction by NPs remains unknown, it has been noted that Fe_3_O_4_ NPs increase the production of reactive oxygen species (ROS) in dermal fibroblasts and cause oxidative stress^82^. Damage caused by ROS in cellular structures can induce autophagy^75^. We have not tested oxidative stress markers in Fe_3_O_4_ NPs treated cells, but induction of autophagy is certainly a cell response to the presence of NP in the culture medium; therefore, the overall role of autophagy induction in NP toxicity should be investigated.

As stated before, toxicity seems to affect internal cellular stress. Since NPs enter cells through endocytosis, various biological processes and enzymes can alter their chemical and physiological properties, making them more toxic upon entry into cells. It is unknown to what extent the modifications introduced are stable in biological structures, but the fact that cells react to all tested NPs, in the same way, suggests that autophagy relies solely on the NP's core. Further research requires studying magnetite nanoparticles' physical and chemical changes inside cells to confirm this.

## Conclusions

Ultrafine, superparamagnetic spherical magnetite nanoparticles functionalized by triethylene glycol or polyethylene glycol were successfully synthesized using the polyol method. It was confirmed that using urotropine significantly improves the colloidal stability of the water dispersions, even at a high concentration level of 10 mg/ml. While the differences in the size of the nanoparticles are negligible (8.69 ± 1.44 nm for the smallest Fe_3_O_4_—NH_4_HCO_3_ and 10.04 ± 1.5 nm for the biggest Fe_3_O_4_ – URO NPs), the magnetically induced hyperthermia effect is dependent on the type of used nanoparticles. This behavior is related to the differences in the aggregate size and zeta potential values. Only Fe_3_O_4_—NH_4_HCO_3_ NPs are characterized by ultrafast temperature growth in all tested frequencies and magnetic field strength, which is highly visible for low *H·f* product values. For 6.6 ∙10^9^ A/ms, these nanoparticles had the highest SAR (69.6 ± 5.2 W/g) value, while Fe_3_O_4_—URO NPs with the highest colloidal stability had SAR equal to 13.4 ± 2.0 W/g. Unfortunately, these nanoparticles synthesized in the presence of NH_4_HCO_3_ cannot be used for cyclic-induced hyperthermia according to the losses in efficiency. These losses were not observed for Fe_3_O_4_—URO NPs, for which the average SAR was equal to 33.20 ± 1.15 W/g.

Moreover, LRT can be applied for low H-field region (up to 17.1 kA/m). Based on this finding, synthesized herein nanoparticles are characterized by higher ILP values than some commercially available dispersions with much higher (even 8 times) concentrations. The cytotoxicity tests show no differences between toxicity on human fibroblasts for all three nanoparticle types, and the highest toxicity was observed for high nanoparticle concentrations and high interaction times with fibroblast cells. Also, ultrastructural analysis confirmed that the nanoparticles do not significantly affect the fibroblasts, excluding a gradual increase in the number of autophagous structures. Moreover, it was concluded that the magnetite nanoparticles synthesized in the presence of different modifiers interact similarly with the cell surface and enter cells by endocytosis.

## Supplementary Information

Supplementary Information.

## Data Availability

The data and material generated during and/or analyzed during the current study are available from the corresponding author on reasonable request.
