# Supplementary material for: Influence of the modifiers in polyol method on magnetically induced hyperthermia and biocompatibility of ultrafine magnetite nanoparticles
Source: Sci Rep. 2023 May 15;13:7860. doi: 10.1038/s41598-023-34738-z (PMC10185678; doi:10.1038/s41598-023-34738-z)
Supplement: Supplementary file 1 — Supplementary Information. [file 41598_2023_34738_MOESM1_ESM.pdf]

Supplementary Materials

Influence of the modifiers in polyol method on magnetically induced hyperthermia and biocompatibility of ultrafine magnetite nanoparticles

Adrian Radoń [1, 2, *], Agnieszka Włodarczyk [3], Łukasz Sieroń [3], Magdalena Rost-Roszkowska [4], Łukasz Chajec [4], Dariusz Łukowiec [1], Agnieszka Ciuraszkiewicz [2], Piotr Gębara [5], Stanisław Wacławek [6], Aleksandra Kolano-Burian [2]

[1] Faculty of Mechanical Engineering, Silesian University of Technology, Konarskiego 18 a St., 44-100 Gliwice, Poland

[2] Łukasiewicz Research Network - Institute of Non-Ferrous Metals, Sowinskiego 5 St., 44-100 Gliwice, Poland

[3] Department of Medical Genetics, Faculty of Medical Sciences in Katowice, Medical University of Silesia, Medyków 18, 40-752 Katowice, Poland

[4] Institute of Biology, Biotechnology and Environmental Protection, University of Silesia in Katowice, Bankowa 9, 40-007 Katowice, Poland

[5] Department of Physics, Częstochowa University of Technology, Armii Krajowej 19, 42-200 Czestochowa, Poland

[6] Institute for Nanomaterials, Advanced Technologies and Innovation, Technical University of Liberec, Studentská 1402/2, 461 17, Liberec 1, Czech Republic

*corresponding author: adrian.radon@imn.lukasiewicz.gov.pl

Table S1. Comparison of the values of aggregates diameter measured using the DLS technique (D_{DLS}) and zeta potential values (ζ potential) determined for the 3 mg/ml water dispersions

Sample	D_{DLS} (nm)	ζ potential (mV)
Fe_3O_4 - URO	120.17±0.34	7.57±0.35
Fe_3O_4 - NH_4HCO_3	272.87±5.77	4.32±1.70
Fe_3O_4 - PEG	395.57±12.60	8.86±1.26

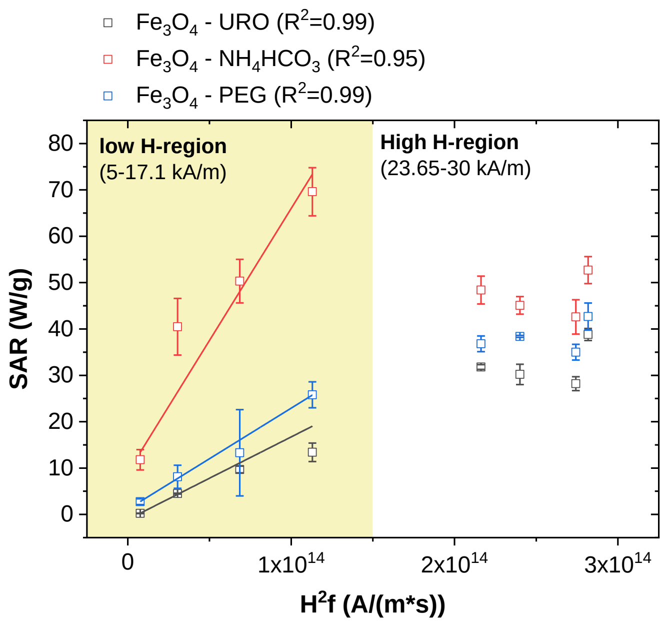

Figure S1. Relationship between SAR and H^2f determined for Fe_3O_4-URO, Fe_3O_4- NH_4HCO_3 and Fe_3O_4-PEG NPs with linear relationship determined for low H-region according to the LRT

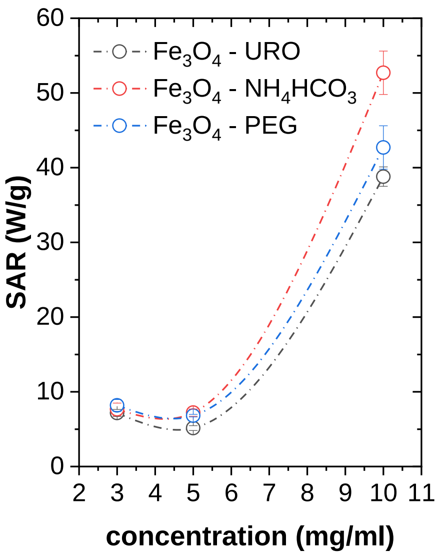

Figure S2. Relationship between SAR and colloids concentrations measured for Fe_3O_4-URO, Fe_3O_4-NH_4HCO_3 and Fe_3O_4-PEG NPs

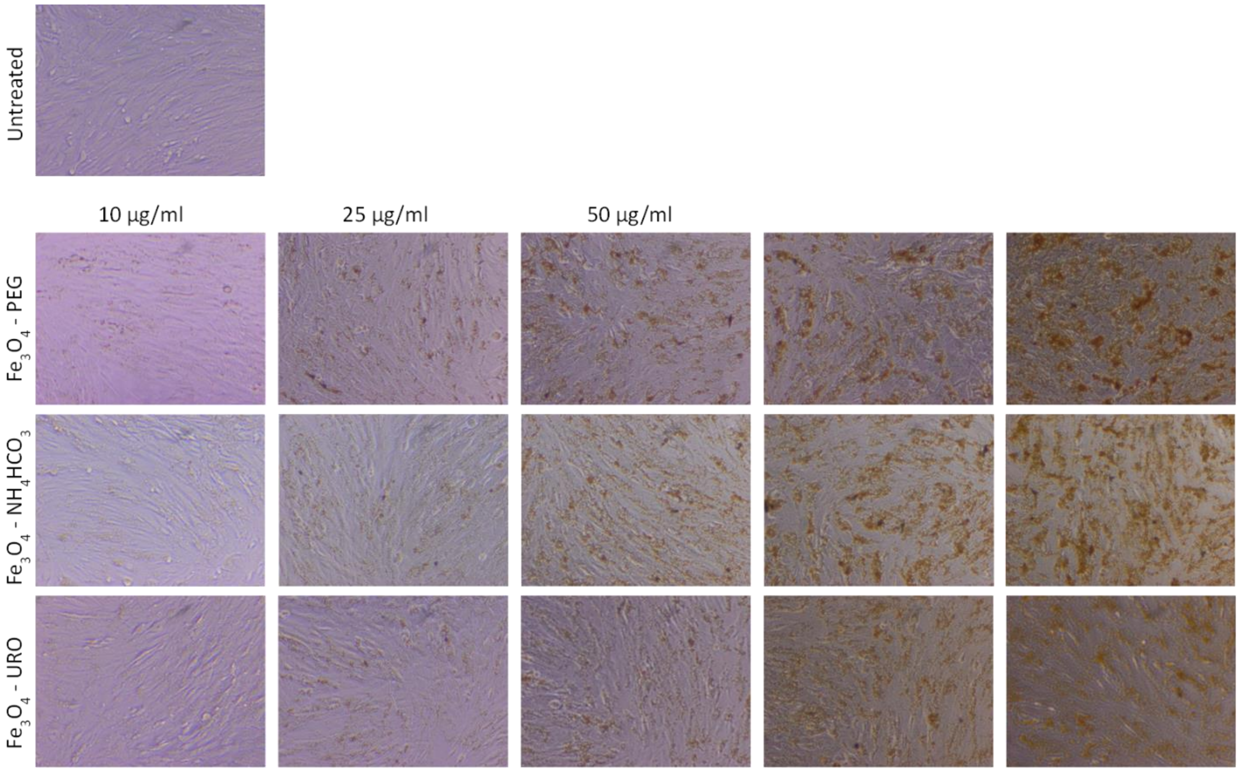

Figure S3. Picture of cells culture from the light microscope. Nanoparticles settled down on the cells layer.

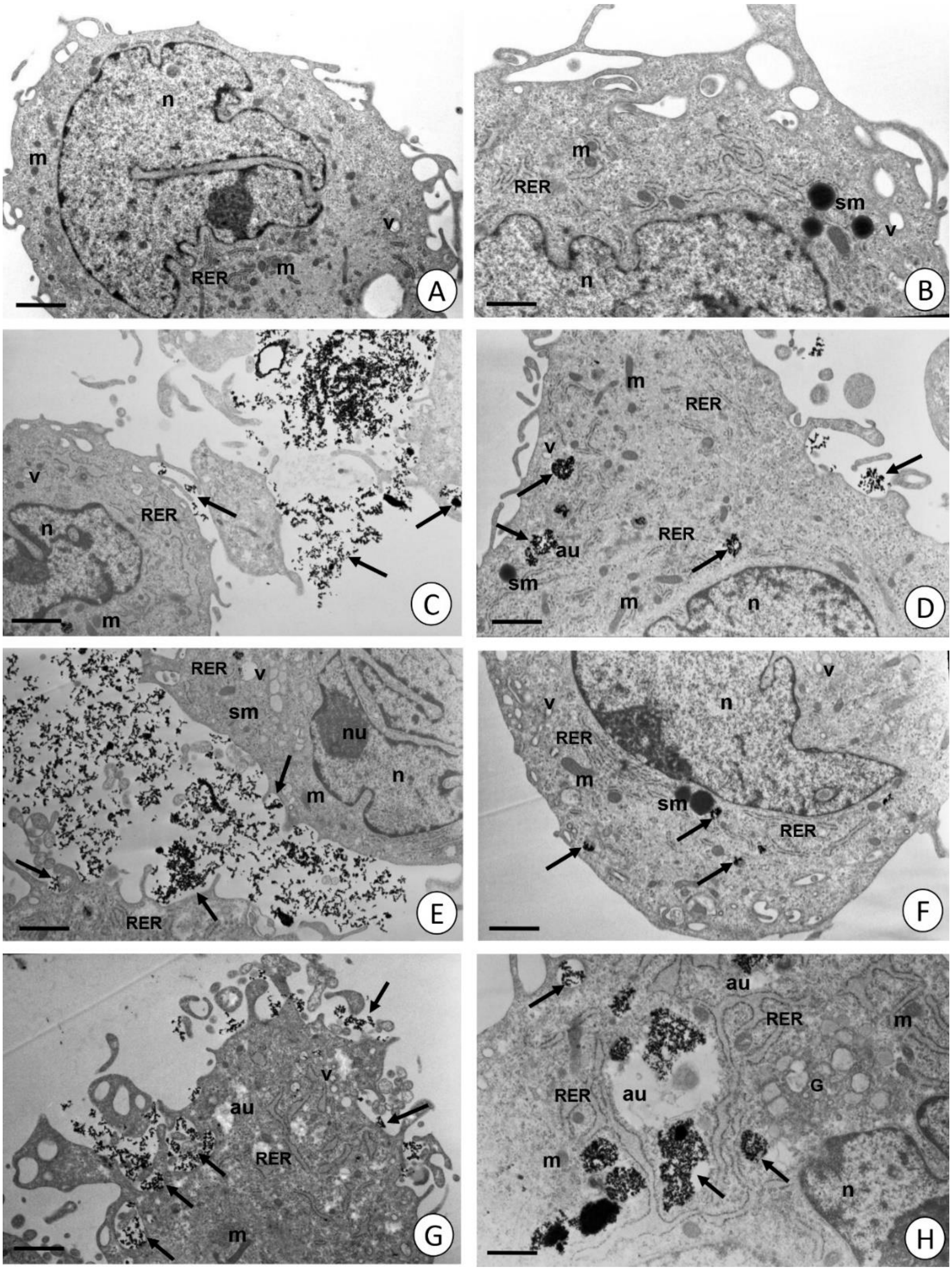

Figure S4. Fibroblasts visible in TEM. **(A-B)** 0 – 24h control group. **(C-D)** Fe_3O_4 **-** PEG – 24h experimental group. **(E-F)** Fe_3O_4 **-** NH_4HCO_3 – 24h experimental group. **(G-H)** Fe_3O_4 **-** URO – 24h experimental group. Nuclei (n), nucleolus (nu), mitochondria (m), cisterns of RER (RER), storage material (sm), Golgi complexes (G), vacuoles (v), autophagosomes (au), electron-dense granules (arrows). **(A)** Scale bar = 1.74 μm. **(B)** Scale bar = 0.87 μm. **(C)** Scale bar = 1.71 μm. **(D)** Scale bar = 1.44 μm. **(E)** Scale bar = 1.66 μm. **(F)** Scale bar = 1.14 μm. **(G)** Scale bar = 1.82 μm. **(H)** Scale bar = 1.04 μm.

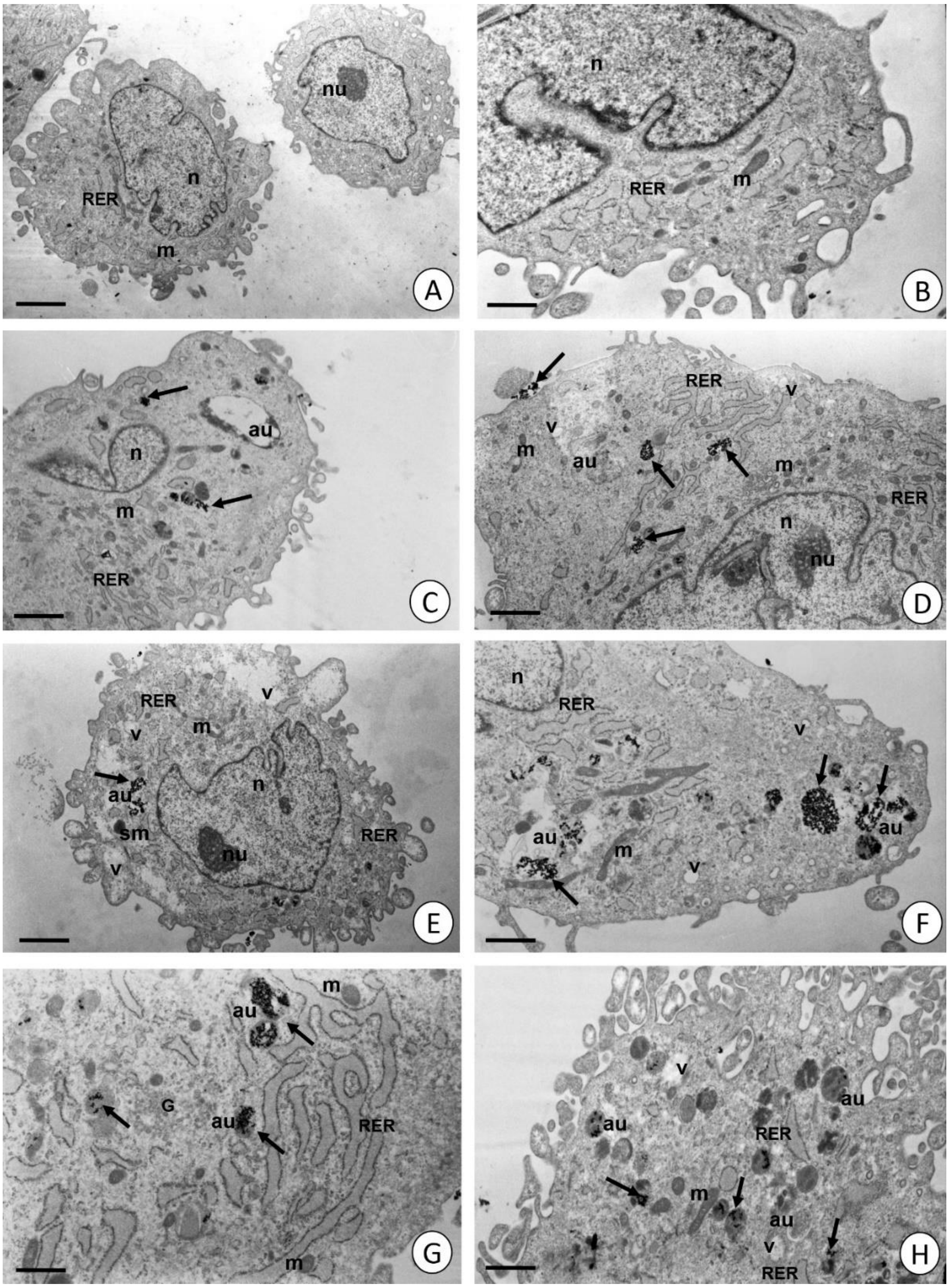

Figure S5. Fibroblasts visible in TEM. **(A-B)** 0 – 48h control group. **(C-D)** Fe_3O_4 **-** PEG – 48h experimental group. **(E-F)** Fe_3O_4 **-** NH_4HCO_3 – 48h experimental group. **(G-H)** Fe_3O_4 **-** URO – 48h experimental group. Nuclei (n), nucleolus (nu), mitochondria (m), cisterns of RER (RER), Golgi complexes (G), storage material (sm), vacuoles (v), autophagosomes (au), electron-dense granules (arrows). **(A)** Scale bar = 2.28 μm. **(B)** Scale bar = 1.10 μm. **(C)** Scale bar = 1.84 μm. **(D)** Scale bar = 1.82 μm. **(E)** Scale bar = 2.28 μm. **(F)** Scale bar = 1.42 μm. **(G)** Scale bar = 1.22 μm. **(H)** Scale bar = 0.88 μm.
